# Uncommon Progression of an Extradural Spinal Meningioma

**DOI:** 10.1155/2014/630876

**Published:** 2014-08-27

**Authors:** Atef Ben Nsir, Mohamed Boughamoura, Houda Mahmoudi, Mohamed Kilani, Nejib Hattab

**Affiliations:** ^1^Department of Neurosurgery, Fattouma Bourguiba University Hospital, Farhat Hached Street, 5000 Monastir, Tunisia; ^2^Department of Pathology, Fattouma Bourguiba University Hospital, Farhat Hached Street, 5000 Monastir, Tunisia

## Abstract

Extradural spinal meningiomas are rare. Our understanding of purely extradural spinal meningiomas is still incomplete and they may be easily confused with malignant neoplasms, much more common in this location. We report a rare case of a purely extradural thoracic spine meningioma in a 70-year-old man, with an unusual progression. In addition we discuss the pathogenesis of these tumors and the potential pitfalls in differential diagnosis and review the relevant literature concerning their treatment and outcome.

## 1. Introduction

Meningiomas account for 25 to 46% of primary spinal neoplasms [1] and spinal meningiomas represent approximately 7.5 to 12.7% of all meningiomas [2]. Extradural spinal meningiomas are infrequent and account for only 2.5 to 3.5% of all spinal meningiomas [3–5]. They show the same histology, the same frequent location in the thoracic spine, and the same well-known sex preponderance of female patients [3].

We report a rare case of a purely extradural thoracic spine meningioma in a 70-year-old man with an uncommon progression. In addition we discuss the pathogenesis of such tumors and the potential pitfalls in differential diagnosis and review the relevant literature concerning their treatment and outcome.

## 2. Case Report

A 70-year-old man with no significant medical history was admitted to our hospital in January 2005 complaining of back pain and sensory changes of both lower limbs for the past 08 months and difficulty in walking for the past two days. He did not present with sphincter disturbances and seemed to be healthy before the onset of the symptoms.

On admission, the patient's neurological examination revealed paraparesis with with motor strength of 3/5, a T6 sensory level and exaggerated knee and ankle reflexes along with extensor plantar response in both sides. There was no bowel or bladder dysfunction.

**Figure 1:**
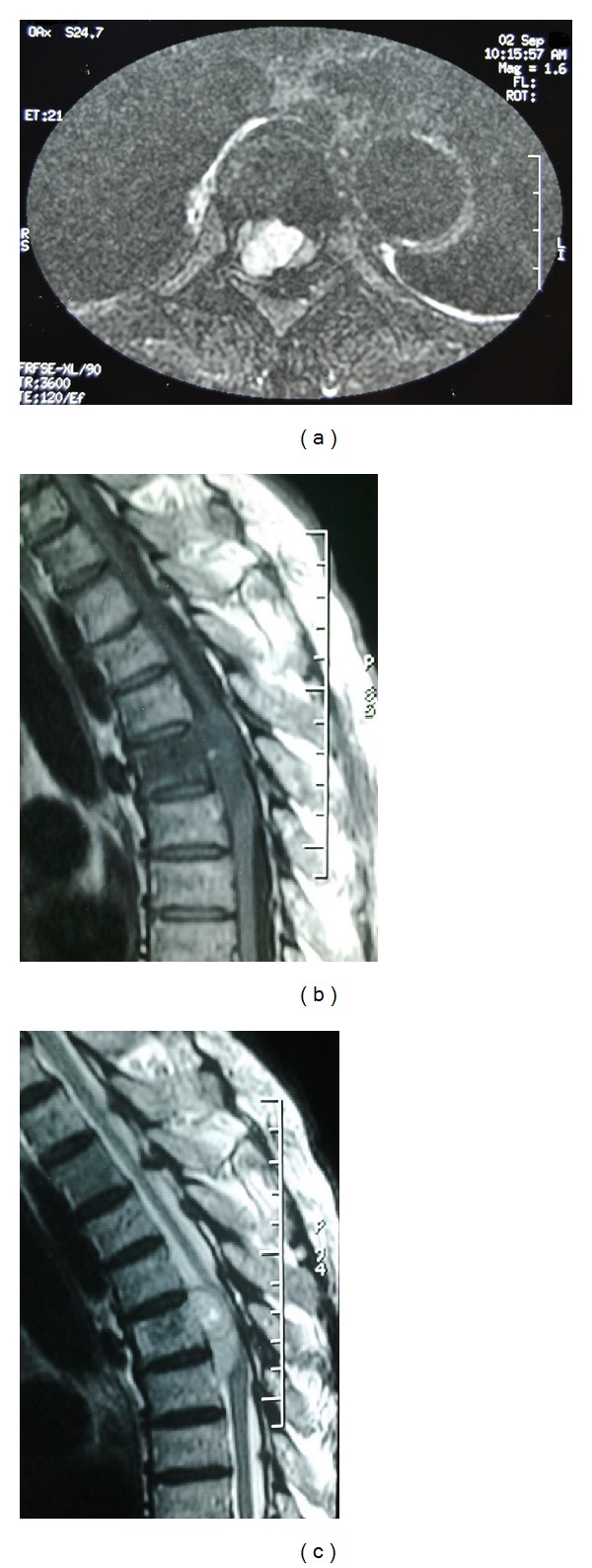
Preoperative axial (a) and sagittal (b) T1-weighted and sagittal (c) T2-weighted MR images through the thoracic spine showing a T1 hypointense, T2 hyperintense, extradural process at T5 level with important spinal cord compression. Note the T5 vertebral body signal changes.

**Figure 2:**
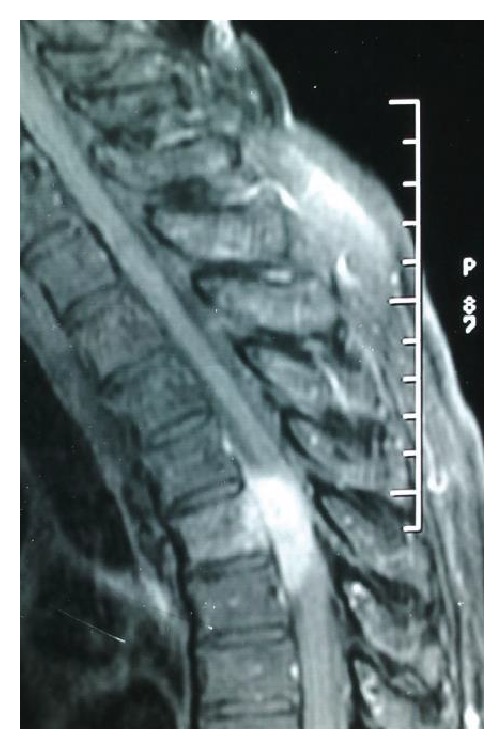
Preoperative sagittal T1-weighted postgadolinium MR image showing important tumor enhancement.

MRI revealed a space occupying anterior lateral extradural lesion at T5 level resulting in marked compression of the thecal sac. The lesion was hypointense to the spinal cord on T1-weighted images, T2 hyperintense, and displayed important enhancement after Gadolinium injection. Vertebral body of T5 was in T2 hypersignal too (Figures 1 and 2).

**Figure 3:**
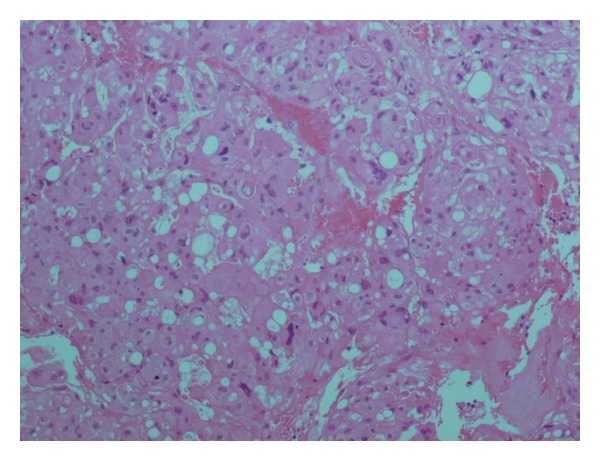
Photomicrographs of the tumor specimen showing lobular architectural pattern with high cellular density and frequent mitosis. Hematoxylin and eosin; magnification: × (200).

**Figure 4:**
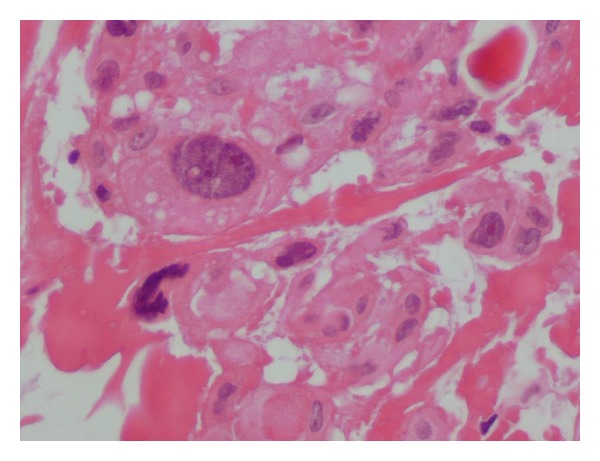
Photomicrographs of the tumor specimen showing nuclear atypia with prominent nucleoli. Hematoxylin and eosin; magnification: × (400).

MRI findings were suggestive of a metastatic extradural and vertebral secondary location but no primary lesion was found in the quick preoperative radiological evaluation. The patient underwent a posterolateral approach in emergency. An anterior lateral, purely extradural, gray tumor was found intraoperatively with an amazing discovery: the tumor was attached to the lateral dural sleeve. The extradural mass was completely excised and the dural basement was cauterized. The definitive histological diagnosis was an atypical (WHO grade II) meningioma as the tumor demonstrated small cell differentiation and increased mitotic activity on light microscopy (Figures 3 and 4).

**Figure 5:**
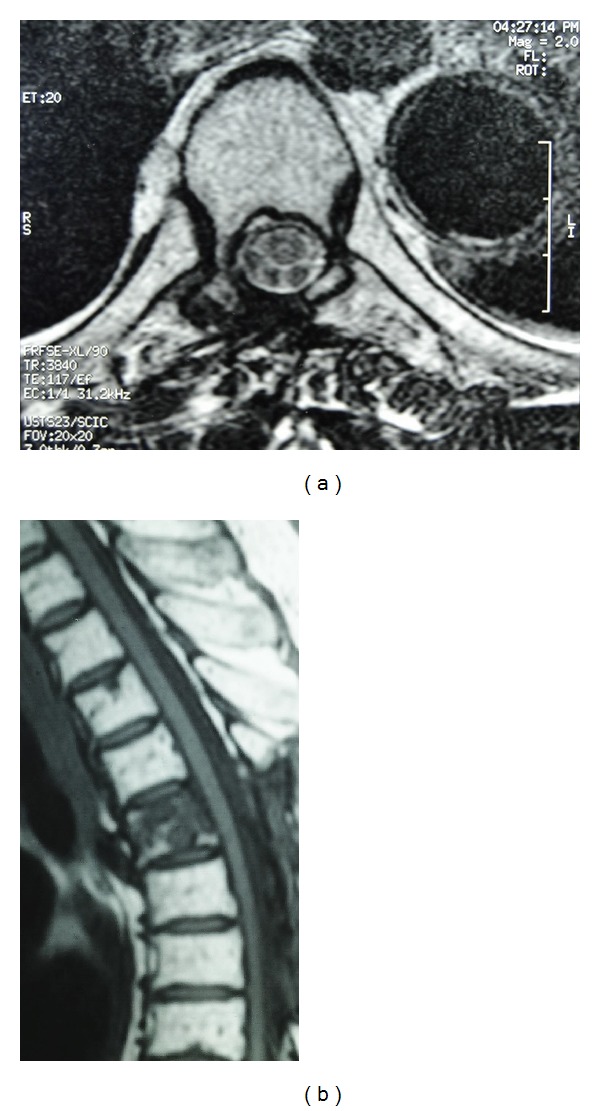
Postoperative axial (a) and sagittal (b) T1-weighted MR images showing extradural tumor removal and spinal cord decompression.

The patient's immediate postoperative course was uneventful, and he recovered quickly. His neurological condition improved continuously, and follow-up examinations showed that the motor weakness had disappeared completely. An adjunctive 50 Gy radiation therapy was administered and six-month serials MRIs were made during postoperative follow-up (Figure 5).

**Figure 6:**
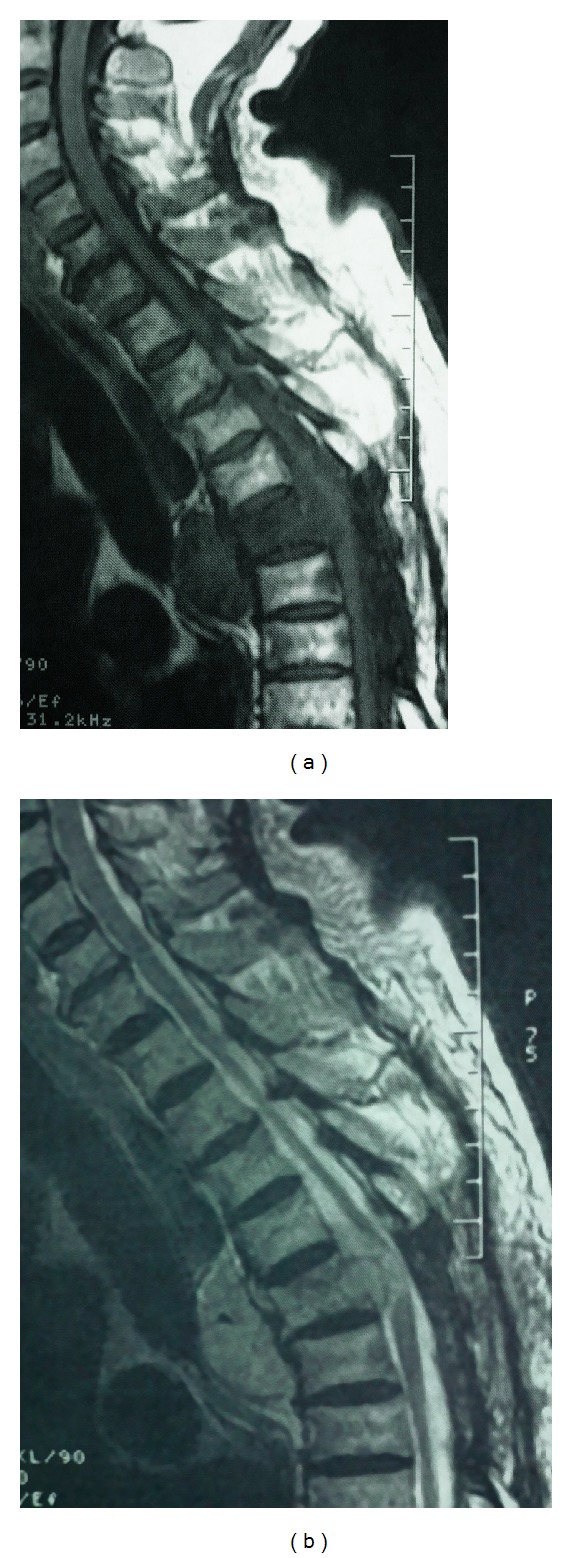
Recurrence sagittal T1-weighted (a) and T2-weighted (b) MR images showing an important space occupying mass with spinal cord compression, T5 vertebral body involvement, and large posterior mediastinum extension.

Two years after surgery the patient was lost to view. He presented once again in February 2012 with severe symptoms of spinal cord compression. MRI showed a local relapse of the disease with mainly anterior lateral progression including T5 vertebral body and posterior mediastinum (Figure 6). A posterolateral transthoracic approach aiming at complete tumor and T5 vertebral body removal and stabilisation was performed. Second histological diagnosis was the same as that during first surgery. Postoperatively, the patient underwent intensive care and rehabilitation with a partial improvement. The patient was able to walk with support with no evidence of recurrence at the most recent follow-up, two years after surgery.

## 3. Discussion

Spinal meningiomas can still cause a great deal of morbidity. They are typically intradural extramedullary and extradural space is usually the site of malignant neoplasms such as metastases or lymphoma especially in the elderly.

Exclusively extradural meningiomas are very rare [1, 4–7]. Furthermore, as many reports use different criteria to distinguish between partially and totally extradural lesions, their real frequency is unknown and may be lower than that already presented. Extradural spinal meningiomas can be easily mistaken pre- and intraoperatively which will certainly interfere with the extent of surgery and affect the patient's outcome. Their origin is still unclear and it is thought that the extradural site might be due to abnormally located meningothelial cells [5].

Purely extradural meningiomas do not essentially differ from the common intradural types [3]. They often affect middle-aged women with an overrepresented female/male ratio compared to intracranial meningiomas (3–4.2 : 1) [3] and are most frequently located in the thoracic spine, less frequently in the cervical spine, and rarely in the lumbar spine [5, 8]. In the present case the purely extradural mass occurred in an old man rather than a middle-aged woman and although a primary lesion was not found in the preoperative radiological investigations, our initial diagnosis was a metastatic tumor.

There is typically a delay between the onset of symptoms and diagnosis. Patients with spinal meningioma often present with pain, sensory motor changes, and sphincter disturbances. Generally back pain precedes the weakness and sensory changes. The sphincter dysfunction is always a late finding [9]. The clinical features of an extradural meningioma, on the other hand, seem to be not different from its intradural counterparts. In our case the clinical presentation was typical and the duration of symptoms prior to presentation was 08 months.

MRI is the best imaging technique for diagnosing spinal meningiomas. It clearly delineates the level of the tumor and its relations to the cord, which is useful in planning surgery [5, 8]. The iso- or hypointensity of this type of lesion on T2 MRI sequences contrasts with the T2 hyperintensity of most epidural tumors, except for lymphomas that can be hypointense in over 50% of cases [1, 5]. In our case the tumor was hypointense to the spinal cord on T1-weighted images, T2 hyperintense, and displayed important enhancement after Gadolinium injection. It was associated with a T2 hyperintensity of the T5 vertebral body. The imaging features are much more suggestive of a metastatic tumor than a meningioma as vertebral body changes are infrequent in spinal meningiomas.

Cushing and Eisenhardt defined the successful removal of a spinal meningioma as one of the most gratifying operative procedures. Posterior laminectomies are indicated for posterior locations. If the tumor is located anteriorly, the laminectomy can be extended laterally towards the articular process to provide sufficient exposure and cause minimal displacement of the spinal cord, or an anterior approach via posterolateral thoracotomy can be indicated. In the present case, a 70-year-old man presented with a rapid neurologic deterioration due to an extradural mass at T5 level. After a brief radiological investigation we decided to achieve a posterolateral approach in emergency with total removal of the extradural mass and cauterization of the dural basement. For the relapse a more aggressive surgical treatment was opted with good surgical results.

Pathologically, spinal meningiomas tend to be well-defined discrete lesions with a dural attachment. Microscopically, the common patterns are meningothelial, fibroblastic, transitional, and psammomatous and most case reports describe features of a meningothelial or psammomatous meningioma [8, 9]. The WHO grades meningiomas in a three-tiered scheme: benign, atypical, and malignant. Our patient's pathology report noted tumor cells of meningothelial subtype with high mitotic index and nuclear abnormalities and the tumour was classified as an atypical (WHO Grade II) meningioma.

In literature review of major spinal meningioma series, including intra- and extradural subtypes, results of surgical excision are reported to be good to excellent with recurrence rates of 3 to 7% [8]. Morbidity and mortality rates are relatively low and complete recovery after tumor removal is common [7, 8] but there is no large series of purely extradural spinal meningiomas. For such condition the literature provides opposing views. In fact, aggressive behavior of these tumors has been asserted by several authors [9, 10]. On the other hand, some investigators believe that extradural meningiomas are usually benign as Roux et al. [4] in their review of 54 cases of intraspinal meningiomas found no significant prognosis differences between intradural and extradural meningiomas.

For the present case, the diagnostic of a malignant lesion was erroneously suspected before surgery and no intraoperative histological examination was performed. We believe it would have been better to completely remove the dural basement of the tumor, or at least one layer of it, to decrease recurrences during the first surgery. Although literature did not provide any guidelines and regard of the dismal progression of our patient's tumor, a second operation would have been necessary after the final pathology results were obtained. Therefore, we want to stress on the importance of intraoperative histological examination for adequate surgical decision making.

Another standpoint is that it is better to reserve adjuvant radiation therapy for recurrent, difficult to reach, high grade cases than after first surgery due to the risk of irradiating a functional spinal cord with no proven benefit, especially that the lesion was anterior and difficult to reach without spinal cord radiation exposure.

Finally, the fact that this patient demonstrated neurologic improvement following his second surgery makes us suggest the possibility of favourable outcome after surgery for recurrent extradural meningiomas.

## Abbreviations

CT: Computed tomography
MRI: Magnetic resonance imaging.
